# Uncertainty of risk estimates from clinical prediction models: rationale, challenges, and approaches

**DOI:** 10.1136/bmj-2024-080749

**Published:** 2025-02-13

**Authors:** Richard D Riley, Gary S Collins, Laura Kirton, Kym IE Snell, Joie Ensor, Rebecca Whittle, Paula Dhiman, Maarten van Smeden, Xiaoxuan Liu, Joseph Alderman, Krishnarajah Nirantharakumar, Jay Manson-Whitton, Andrew J Westwood, Jean-Baptiste Cazier, Karel G M Moons, Glen P Martin, Matthew Sperrin, Alastair K Denniston, Frank E Harrell, Lucinda Archer

**Affiliations:** 1 Department of Applied Health Sciences, School of Health Sciences, College of Medicine and Health, University of Birmingham, Birmingham, UK; 2 National Institute for Health and Care Research (NIHR), Birmingham Biomedical Research Centre, Birmingham, UK; 3 Centre for Statistics in Medicine, Nuffield Department of Orthopaedics, Rheumatology and Musculoskeletal Sciences, University of Oxford, Oxford, UK; 4 Cancer Research UK Clinical Trials Unit, School of Medical Sciences, College of Medicine and Health, University of Birmingham, Birmingham, UK; 5 Julius Center for Health Sciences and Primary Care, University Medical Centre Utrecht, Utrecht University, Utrecht, Netherlands; 6 Department of Inflammation and Ageing, School of Infection, Inflammation and Immunology, College of Medicine and Health, University of Birmingham, Birmingham, UK; 7 Peterhouse, Trumpington Street, University of Cambridge, Cambridge, UK; 8 Bone Cancer Research Trust, Leeds, UK; 9 Francis Crick Institute, London, UK; 10 Division of Informatics, Imaging and Data Science, Faculty of Biology, Medicine and Health, University of Manchester, Manchester Academic Health Science Centre, Manchester, UK; 11 Department of Biostatistics, Vanderbilt University School of Medicine, Nashville, TN, USA

## Abstract

Clinical prediction models estimate an individual’s risk (probability) of a health related outcome to help guide patient counselling and clinical decision making. Most models provide a single point estimate of risk but without the associated uncertainty. Riley and colleagues argue that this needs to change, as understanding uncertainty of risk estimates helps to inform critical evaluation of a model and may impact shared decision making. Examples are provided to illustrate uncertainty in risk estimates, and key methods to quantify and present uncertainty are discussed.

## Introduction

**Fig 1:**
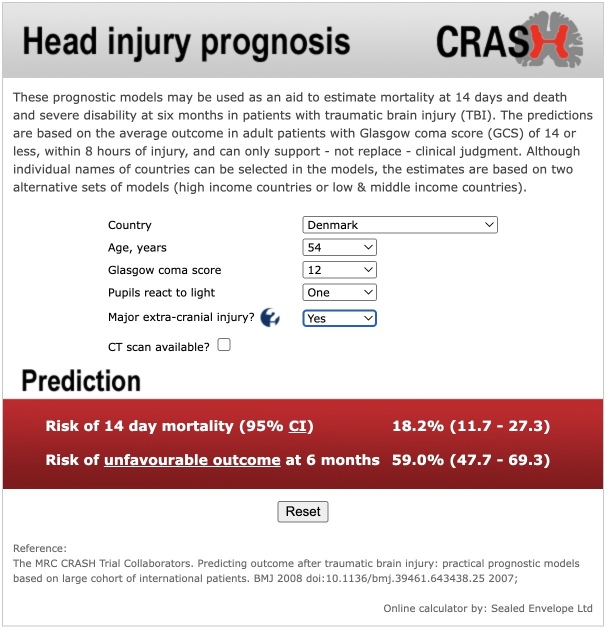
A screenshot of the output from the webtool of the CRASH prediction model when applied to a hypothetical individual. The CRASH models are logistic regression models that estimate the risks of 14 day mortality and six month unfavourable outcome (death or severe disability) in patients with traumatic brain injury. The output includes a point estimate of risk (expressed as a %) and the corresponding 95% uncertainty interval (labelled as confidence interval (CI)) (see www.crash.lshtm.ac.uk/Risk%20calculator/index.html)

Each year, thousands of clinical prediction models are published in the medical literature aiming to inform diagnosis or prognosis in a particular target population.^1^ They are used to estimate an individual’s risk (probability) of having (diagnosis) or developing (prognosis) a health related outcome conditional on their values of multiple predictors, to help guide patient counselling and clinical decision making. Examples include QRISK3,^2^ which is widely used in the UK during primary care consultations to estimate a person’s 10 year cardiovascular disease risk, to inform lifestyle changes or statin prescribing in people deemed to be at high risk; and the IMPACT and CRASH tools that estimate a patient’s risk of mortality and other adverse outcomes following hospital admission for a traumatic brain injury.^3^ ^4^ For example, figure 1 shows an application of the CRASH tool to a hypothetical 54-year-old. Based on the individual’s particular characteristics (predictor values), the model’s point estimate (best guess) of their risk of an unfavourable outcome by six months is 0.59. This value is presented as 59% because some researchers convert risks (probabilities) on the 0 to 1 scale to percentages on the 0 to 100 scale for dissemination purposes; in this article, we prefer to use the 0 to 1 scale. We also use the terms risk and probability interchangeably.

Clinical prediction models are developed using regression approaches (eg, logistic regression) or methods attributed to artificial intelligence (AI) and machine learning (eg, tree-based methods and deep learning), all of which map predictor values to clinical outcomes at the individual level. A model should be critically appraised and rigorously evaluated before being considered appropriate for use in clinical practice.^5^ ^6^ ^7^ Unfortunately, the vast majority of published models are not suitable for clinical use due to poor methodological standards such as small sample sizes,^8^ ^9^ inappropriate model development techniques, and little (external) evaluation in new data.^10^ For example, a review of 606 Covid-19 prognostic models identified that 545 were at high risk of bias,^11^ such that their reported predictive accuracy is likely optimistic, and estimated outcome risks poorly calibrated with observed outcome risks.

A key aspect of reliability is model stability^12^: if a different sample of the same size was used from the same overarching population, how different would the developed model and its predictions be? This issue motivates our article, and is sometimes referred to as epistemic (reducible) uncertainty and is caused by sampling variability during model development.^13^ To expose any instability, an indication of the uncertainty of model predictions is helpful; for example, by presenting an individual’s point estimate of risk alongside a 95% uncertainty interval (eg, corresponding to a confidence interval, following a frequentist analysis; or a credible interval or highest posterior density interval, following a Bayesian analysis) or an uncertainty distribution (eg, derived using bootstrapping in a frequentist analysis^12^; or the posterior distribution from a Bayesian analysis). For example, based on the CRASH model application in figure 1, a 95% uncertainty interval for this individual’s risk of an unfavourable six month outcome is 0.477 to 0.693 (presented as 47.7% to 69.3%). The range of values in the interval are all consistent with the individual having a high risk of an unfavourable outcome by six months. However, if the interval covers a wide range of risks (eg, 0.081 to 0.891), whether the model provides sufficiently precise information to inform clinical decisions for that individual might be in doubt.

Much inconsistency and debate surround whether uncertainty in risk estimates should be presented and how this can be done in practice.^14^ Some people argue in favour of presenting uncertainty,^15^ ^16^ others argue against,^17^ ^18^ but generally, uncertainty around risk estimates is ignored. For example, IMPACT is another prognostic model in traumatic brain injury but, unlike the CRASH tool, it does not output uncertainty intervals alongside point estimates of risk (www.tbi-impact.org/?p=impact/calc).

In this article, we recommend that clinical prediction models *should* indicate the uncertainty of their predictions, even when single point estimates are intended to guide clinical decision making. Our focus is on models that estimate risks for individuals. We discuss reasons why quantifying and presenting uncertainty of risk estimates helps to better inform those critically appraising a model and those potentially using it, including doctors and patients. Furthermore, we show that accounting for uncertainty might even change an individual’s point estimate of risk itself. We draw on conversations from patient and public involvement and engagement (PPIE) groups, and emphasise the potential challenges of communicating and interpreting uncertainty of risk. To help researchers who are developing and evaluating prediction models, we conclude by outlining some key methods to quantify uncertainty of predictions using model development and evaluation datasets.

## Reasons why presenting uncertainty of risk estimates is important

We outline five key reasons why presenting the uncertainty of risk (probability) estimates can be beneficial. The reasons are interrelated but mentioned separately to help reinforce the argument.

**Fig 2:**
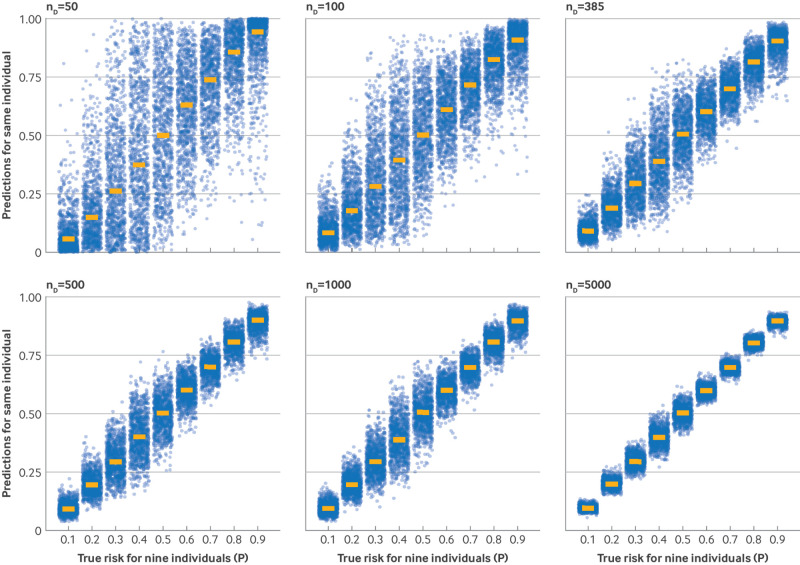
1000 risk estimates (“predictions”, y axis) sampled from the uncertainty distribution for nine individuals (with true risks (P), x axis, between 0.1 and 0.9), across six different models developed in sample sizes (n_D_) of 50, 100, 385, 500, 1000, and 5000 participants. Each model was produced by fitting a lasso logistic regression to a different random sample of individuals simulated from the same population with a true overall risk of 0.5, considering one genuine predictor (X∼N(0,4)) and 10 noise variables (Z1,…, Z10∼N(0,1)). Figure adapted from Riley and Collins with permission.^12^ The smaller the sample size, the wider the uncertainty distribution, even spanning the entire range of 0 to 1 in small samples.

Firstly, and fundamentally, expressing uncertainty of an individual’s outcome risk provides a more complete picture than just a point estimate, as the sampling variability (or stability^12^ ^13^) of a model’s prediction is shown. A point estimate of an individual’s risk is a single value (best guess); for example, an individual’s point estimate might be calculated as the average value of the model’s uncertainty distribution for their risk. However, providing the entire uncertainty distribution shows other plausible values, potentially indicating a wide range of possible risks for an individual. Sampling variability and model instability generally increase with smaller model development sample sizes (lower numbers of participants and outcome events), larger numbers of candidate predictors for inclusion in the model, and low signal to noise situations (ie, smaller R^2^).^12^ ^19^ Variability is hidden when only a point estimate of risk is reported. Figure 2 illustrates this variability for models based on simulated data,^12^ with the uncertainty of individual risk often spanning the entire range of 0 to 1 in smaller sample sizes. Therefore, quantifying uncertainty in model predictions provides a useful model performance metric to be presented alongside other aspects, including whether risk estimates are well calibrated in the overall population and key subgroups.^20^ Expressing uncertainty is consistent with other areas of medical research, for example, in randomised trials, where uncertainty of estimates are expected to be presented (eg, 95% confidence intervals around treatment effect estimates).

Secondly, understanding uncertainty of a model’s predictions may help health professionals, organisations, and policy makers to decide whether to use or endorse that model, and identify when further information or research is needed.^21^ That is, although a model’s point estimate of risk provides the model’s best guess to inform individual level decisions (eg, within a decision analysis framework, see supplementary material S1), the corresponding uncertainty gives insight about the strength of evidence behind the model informing that decision. Vickers and colleagues support this argument,^22^ noting that “decision analysis tells us which decision to make for now, but we may also want to know how much confidence we should have in that decision. If we are insufficiently confident that we are right, further research is warranted.” For example, if risk thresholds are used to guide a particular clinical action (eg, initiate treatment if an individual’s point risk estimate is ≥0.05), the proportion of an individual’s uncertainty distribution that falls on either side of the risk threshold could be calculated. If this is deemed inconclusive in the clinical context at hand (eg, the percentage below (above) is 60% (40%)), this might motivate obtaining further information to better inform the clinical action.^23^

Thirdly, expressing the uncertainty of risk estimates from a model will inform those performing critical appraisal and quality assessment of the model. For example, peer reviewers, journal editors, systematic reviewers, regulators, and those working for bodies such as the National Institute for Health and Care Excellence (NICE) or the World Health Organization (WHO) often review the evidence about a model’s performance to judge whether they should be recommending it for publication or endorse it in clinical guidelines. If it were known that a model gives estimated probabilities with large uncertainty (eg, due to a small sample size at model development^19^), this model should be flagged as high risk of bias, especially if no high quality external validation studies are available. Thereby, expression of uncertainty informs completion of the Prediction model Risk Of Bias Assessment Tool (PROBAST) and helps apply the Grading of Recommendations Assessment, Development, and Evaluation (GRADE) system to prediction models.^24^ ^25^

Fourthly, understanding uncertainty of a model’s predictions helps contribute towards assessments of whether the model may be unfair or inequitable in some subgroups.^26^ This assessment is important as part of a model’s fairness checks^27^ to assess whether the reliability (accuracy) of predictions is acceptable for all patient groups, including minoritised and underserved groups, not just in the population as a whole.^28^ If a model has large uncertainty of risk estimates for a particular group, then the data might not useful for that group. This issue is hidden if only point estimates of risk are provided and may lead to very uncertain (potentially misleading) risk estimates being used in some individuals. Net harm might be possible if, for example, individuals are not flagged for appropriate treatment or monitoring. Risk estimates from a model will be more uncertain for individuals who were inadequately represented in the development dataset,^29^ specifically individuals with less common combinations of predictor values (ie, rare characteristics).

Lastly, within shared decision making, patients may ask health professionals how sure they are about the evidence (including risk estimates) being used to inform decisions. In such situations, information about uncertainty (and other quality of evidence information) should be readily available to inform the doctor-patient conversation as appropriate. Being able to communicate that a model’s predictions are precise (and well calibrated) for an individual’s personal characteristics may improve the patient’s confidence and trust in using the model to inform decisions, which may reduce anxiety and enhance management of their condition, including improved treatment concordance and monitoring adherence. Conversely, expressing that a model’s estimated risk is very uncertain for a patient’s characteristics may justify shared decisions to abstain from using that model entirely,^16^ and motivate patients requesting other information that is more reliable alongside expert clinical opinion. We return to the challenge of medical communication later.

### Perspective from PPIE groups

Our recommendation that clinical prediction models should quantify and present the uncertainty of their predictions also stems from conversations held with PPIE groups. In particular, during our STANDING Together initiative (aiming to establish STANdards for data Diversity, INclusivity and Generalisability in healthcare AI),^28^ PPIE groups expressed that generally they would want the uncertainty in their own prediction to be communicated to them, to help them make a personal decision in the context of available options. This conclusion was reinforced by a PPIE group for Ewing sarcoma, who told us that clinicians should discuss with their patients that “this is the most likely case for you, but the most likely case still has [particular uncertainty] around it.”

In addition, when we gave an example of how an individual’s estimated risk impacts treatment decisions, the Ewing sarcoma group concluded that uncertainty intervals should be a part of the doctor’s explanation for treatment choices in practice. One representative noted that they considered providing patients with all the knowledge available about a model and its performance “ethically mandatory”, including the uncertainty of predictions, if they request it. A transcript of this representative’s view is provided in Box 1.

Box 1 **Transcript of thoughts from a member of a patient and public involvement and engagement group for people with Ewing Sarcoma**

“When I think back to the comment from our consultant that [patient] had a “70% chance of survival”, I now wonder how reliable that figure could possibly have been. Had it been possible to tell us what was the uncertainty interval around that figure, it might have led to changes in [their] treatment. It would certainly have left us feeling better informed and given us a chance to think about the trade-offs between efficacy of treatment and the unwanted side effects that can arise. Even now, I am not sure that this data exists, and how reliable that 70% figure is at the individual level…

.. Patients and their families are often significantly under-informed on the relative merits and downsides of treatment. Most will rely on their physician's advice and guidance, so even if patients themselves do not have access to the predictive models or have them explained (as would be desired), doctors certainly should.

I believe in the old adage, knowledge is power. To make patients aware of the inherent uncertainty in any predictive model is, to my mind, ethically mandatory. To give them the opportunity to get some idea of how uncertain the prediction is, is arguably just as important. I think most patients want to be given the best available information, and to have explained to them how that information may, or may not, be completely useful in their particular case.

However, I do acknowledge that there are instances when patients/parents may not wish to be given this additional information. Some may choose not to access it. Some may reason that the knowledge would not change their decisions. If there is only one treatment path available, does it matter if you have absolute or only partial confidence of the outcome? That is ultimately a decision for the patient.”

However, this transcript also highlights that communicating uncertainty of risks is a complex issue and will not always be appropriate because the clinical context alongside each individual’s needs and level of understanding is variable. We return to this issue later in the article (see “challenges in communicating and interpreting uncertainty of risk estimates”).

## Accounting for model uncertainty when deriving point estimates of risk

Incorporating model uncertainty is important when deriving an individual's risk estimate, as the estimate may vary depending on its inclusion. In particular, a typical regression based model derives an individual’s point estimate from a fixed model equation (eg, based on the estimated parameters in a logistic regression) that ignores any uncertainty in the model parameters. However, to account for model uncertainty, deriving an individual’s uncertainty distribution for their risk and then calculating their point estimate directly from that distribution is preferable. For example, an individual’s point estimate could be taken as the mean (expected) value of their uncertainty distribution. This is akin to calculating the mean of the individual’s posterior distribution for their risk when the (regression) model is fitted in a Bayesian framework; or the mean of the distribution of risk estimates obtained from a bootstrap process in a frequentist framework (see later). Other point estimates may also be relevant, such as the median or the mode of the uncertainty distribution.^30^

**Fig 3:**
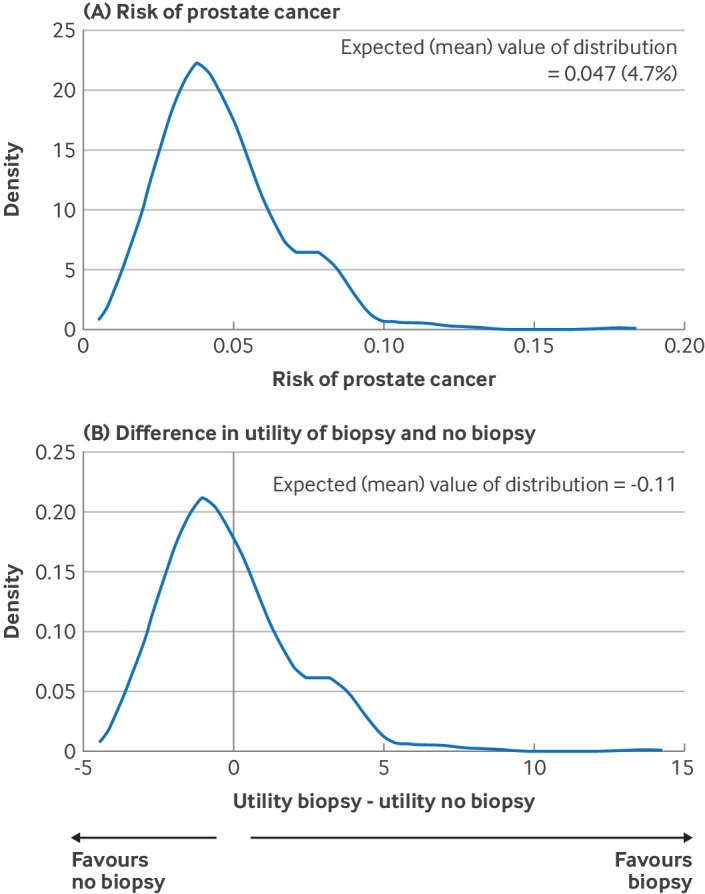
Uncertainty distributions derived from bootstrapping for a particular individual after fitting a logistic regression model to estimate risk of having prostate cancer. Based on the model, panel A shows their risk of prostate cancer and the bottom panel shows the difference in their utility of choosing biopsy or no biopsy. The difference in utility is zero if their risk of prostate cancer is 0.05 (5%), as this is the individual’s chosen threshold for biopsy (see supplementary material S1). When ignoring uncertainty in the estimated model parameters, the individual’s point risk estimate is 0.051 (5.1%) and their expected utility is higher for biopsy than no biopsy. By contrast, when uncertainty is accounted for, panel A shows their point (mean) risk estimate is 0.047 (4.7%), as this is below the individual’s chosen risk threshold of 0.05 (5%), it suggests no biopsy is the preferred decision. Panel B has an expected (mean) value of distribution of −0.11. As this is negative, no biopsy is suggested as the preferred decision

Consider a diagnostic prediction model to estimate the risk of having prostate cancer for a particular individual who specifies that, if their risk is ≥0.05, they would choose to biopsy. Applying the fitted model (a logistic regression) equation ignoring uncertainty in the model parameters, the individual’s point estimate of risk is 0.051 and so, as this value is above their threshold, the decision would be to biopsy. However, use of the actual uncertainty distribution for the individual’s risk, which is shown in figure 3A, is more accurate; this distribution has a mean (median) value of 0.047 (0.043), which suggests not to biopsy because this value is below their threshold. Thus, although the difference in point estimates is small (eg, mean is 0.047 compared with a point estimate of 0.051 derived from the logistic equation), the more exact approach, accounting for uncertainty, has the potential to change the individual’s decision here (based on the model alone).^22^ This issue is most likely to occur in individuals with point estimates that are close to their chosen risk threshold, and with heavily skewed uncertainty distributions. Supplementary material S1 shows this formally using a decision analysis framework,^31^ leading to a higher expected utility for no biopsy compared with biopsy after accounting for uncertainty (figure 3B).

## Challenges in communicating and interpreting uncertainty of risk estimates

As outlined previously, understanding uncertainty of risk estimates is important for different stakeholders including model developers, health professionals, and those critically appraising a model. However, acknowledging uncertainty does raise potential challenges for medical communication, for example, in the doctor-patient consultation, and may not always be appropriate. Indeed, we do not recommend that uncertainty of risks is always (ie, by default) presented and communicated to patients. Furthermore, even when it is appropriate, any communication of uncertainty needs to be tailored for the setting and individual at hand, for the following reasons.

Firstly, a single point estimate of risk can often be difficult to communicate and interpret for some end users of the model. For example, if a model estimates an individual’s five year risk of death to be 0.3, the health professional may relay this value to the individual as: “In a group of 100 individuals with the same characteristics as you, based on the model, we would expect 70 of them to be alive at five years, and 30 not to be alive at five years.” In this statement, uncertainty is already present because we do not know if the individual will be one of the 70 who will be alive, or one of the 30 who will not. Thus, expressing additional uncertainty around the 0.3 risk estimate adds an extra challenge for the doctor-patient consultation, which may often be an unnecessary complication.^17^ ^18^

Secondly, concerns have also been raised that communication of uncertainty might increase patient anxiety and reduce trust in health professionals.^32^ For example, International Patient Decision Aids Standards collaboration recommends healthcare professionals to be cautious about presenting uncertainty of risk estimates^33^ because “this uncertainty may be psychologically aversive and difficult to understand, and that optimal methods of communication remain to be determined.” Furthermore, Politi and colleagues performed a review of communicating uncertainty of harms and benefits of medical interventions,^32^ and conclude that “both patients' and physicians' interpretation of and responses to uncertainty may depend on their personal characteristics and values and may be affected by the manner in which uncertainty is communicated.” Therefore, a patient-specific approach may be required when considering communicating uncertainty of risk estimates from prediction models, especially because not all patients will want or benefit from this information (box 1). Further research into communication of uncertainty is needed.

Finally, care is needed when interpreting whether the extent of uncertainty makes a model’s risk estimate unacceptable for a particular individual, as this acceptability is context and individual dependent, potentially depending on any personal risk thresholds for their decision making.^34^ For example, returning to the prostate cancer example, if a model estimates an individual’s risk of prostate cancer to be 0.005 (0.5%), but with a 95% uncertainty interval of 0.001 to 0.10 (0.1% to 10%), the impact of this interval depends on the specific individual. If they are aged 85 years with many existing comorbidities, they might conclude the interval range reflects a low enough risk to justify no biopsy. By contrast, a younger man aged 40 years with no comorbidities, for whom interventions may substantially prolong life if prostate cancer is detected early, might be concerned about the upper range risk of 0.10 (10%) and request additional information to better inform their decision.

## How to quantify uncertainty of individual risk estimates from prediction models

We now describe methods to derive uncertainty of risk estimates when using model development and evaluation (validation) datasets. We focus on the key statistical approaches and do not intend to be exhaustive; the topic is an area of growing methodological research with emerging approaches gaining interest, such as conformal prediction.^35^ ^36^ A detailed overview is provided by Kompa and colleagues.^16^ When quantifying uncertainty, datasets should be used that are high quality (eg, with appropriate methods of measuring outcomes and predictors)^37^ and representative of the target population and setting where the model will be applied in practice. If this is not the case, then using datasets to estimate uncertainty of individual risk (or indeed a point estimate of risk) may not be reliable.

### Deriving uncertainty of risk estimates using the model development dataset

After fitting a model in a Bayesian framework, the uncertainty of an individual’s risk is naturally summarised by their posterior distribution of risk conditional on their predictor values and all parameter uncertainties in the fitted model. For example, Fanconi and colleagues used a Bayesian framework to fit logistic regression models with the aim to estimate the risk of acute care use in patients with cancer after starting chemotherapy.^38^ They derived posterior distributions for each individual’s risk, which were used to identify individuals with uncertainty intervals that overlapped a chosen risk threshold of 0.2, for whom the authors suggested further information is required before classification (this can also be presented using a classification instability plot^12^). They also compared uncertainty for different ethnic subgroups, as part of model fairness checks, and identified that Black individuals had higher uncertainty in their predictions than people of Asian, White, or of another ethnic group.

**Fig 4:**
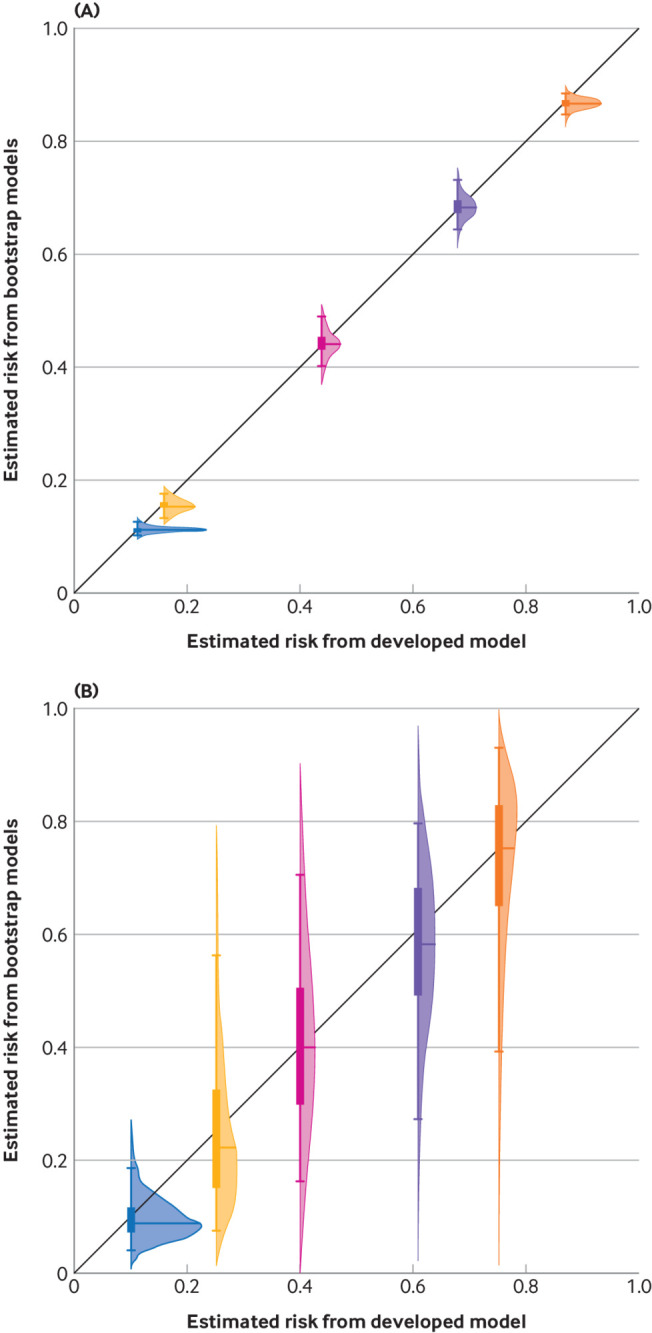
Uncertainty intervals and distributions produced by applying the bootstrap process to models developed with large (40 830 participants, top) and small (500 participants, bottom) datasets. We developed a prediction model to estimate the risk of 30 day mortality in individuals diagnosed with an acute myocardial infarction, using the GUSTO-1 dataset.^41^ A lasso logistic regression model was fitted considering eight predictors, as described elsewhere,^13^ firstly using (panel A) the full sample of 40 830 participants (2851 deaths) referred to as Model A; and (panel B) a random subsample of 500 participants (35 deaths) referred to as Model B. After fitting each model, we applied the bootstrap process (using 10 000 bootstrap models) to derive uncertainty distributions and intervals for the same five individuals. Intervals are defined between capped lines (95%) and coloured boxes (50%).

In a frequentist framework, after fitting a standard (unpenalised) regression model (eg, logistic regression with the CRASH tool) the variance-covariance matrix of the parameter estimates (intercept and predictor effects) can be used to derive uncertainty intervals and distributions.^39^ A more general approach is bootstrapping,^12^ ^40^ which is described in supplementary material S2. Figure 4 uses the bootstrap process to obtain uncertainty intervals and distributions for two comparable models (panel A (model A) and panel B (model B)), both developed using logistic regression with a lasso penalty (to address potential overfitting) and applied to the same five individuals, where the aim was to estimate the risk of 30 day mortality in individuals diagnosed with an acute myocardial infarction. Model A was developed using a large dataset,^41^ and Model B was developed using a small dataset. Model B was far more unstable due to the small sample size used for development, and thus gave less reliable point estimates reflected by wide uncertainty intervals and distributions. As such, Model B could not be used to inform decisions for some individuals. For example, one individual had a point estimate of 0.24 from Model B, but a 95% uncertainty interval of about 0.08 to 0.58, ranging from quite low to very high risk.

The bootstrap approach has a key advantage of being able to be applied to any model development method (eg, penalised regression, random forest, or deep learner) and outcome data type (eg, binary or time-to-event). This method can also account for any predictor selection steps and thus gives a better reflection of the uncertainty than if just based on the final set of predictors. Example code for using bootstrapping is provided at https://github.com/gscollins1973/Instability and elsewhere.^12^ However, the steps can be computationally intensive when using big datasets, deep learning methods, or multiple imputation to handle missing data, for example.

Crucially, regardless of the method used to quantify uncertainty, the actual model development approach must target well calibrated predictions (ie, estimated and observed risks should agree, ideally across the full spectrum of risks from 0 to 1). Otherwise, uncertainty distributions will reflect the uncertainty in predictions that are poorly calibrated in the population, which is not helpful. For example, the model development process could include an (additional) bootstrap or cross-validation process to check and adjust for any miscalibration as part of model tuning, or use a hold-out calibration dataset as in conformal prediction approaches,^16^ which is similar to when evaluating a model in a new dataset, as follows.

### Deriving uncertainty of risk when evaluating models in test or evaluation datasets

Currently, when evaluating an existing model in new data, the uncertainty of that model’s predictions is difficult to take forward from the development dataset. This challenge is because most models only allow users to calculate (eg, via a regression equation, web tool, or mobile app) a single point estimate of risk for an individual. For uncertainty to be carried forward, the existing model would need to provide additional information from the development stage, such as all 1000 bootstrap models (supplementary material S2), the variance-covariance matrix of parameter estimates,^39^ or the original development dataset itself with code to enable derivation and sampling from an individual’s uncertainty (posterior) distribution of risk. We hope provision of this information becomes common practice,^42^ but until then, researchers evaluating a model will need to derive uncertainty of risks based on the evaluation (test, validation) dataset itself.

When using an evaluation dataset, deriving uncertainty of risks fully conditional on all predictor values in the original model is usually difficult, but examining uncertainty conditional on estimated risk is possible by using calibration plots and calibration curves. Calibration examines the agreement between estimated risks (from the existing model) and observed risks (in the evaluation dataset), and the uncertainty in calibration performance stems entirely from the number of participants and observed outcome events, and the participants’ distribution of risk estimates, in the evaluation dataset itself.

**Fig 5:**
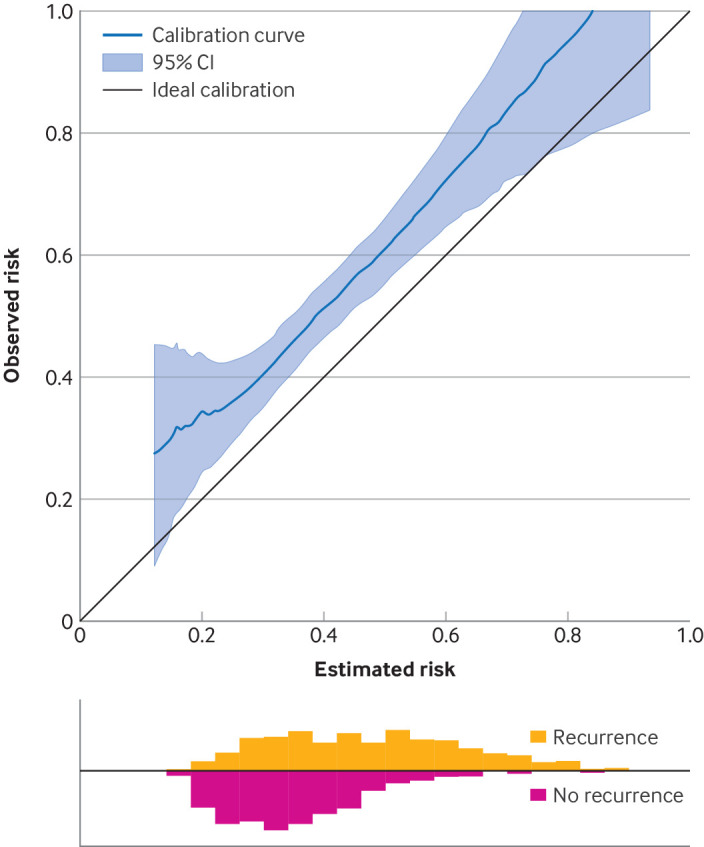
Example of a calibration plot containing a smoothed calibration curve and its 95% confidence interval, from an external validation of a model to estimate five year recurrence risk after a primary breast cancer diagnosis; modified from Riley et al with permission.^7^ Histograms beneath the plot show the distribution of estimated risks for those with and with no recurrence by five years. Example code to generate this plot is available from https://www.prognosisresearch.com/software. CI=confidence interval

Evaluation datasets must contain the values of the outcome, and any predictors used in the model, so that the model can be applied to every individual (ie, to make predictions) and comparisons made between predicted and observed outcomes. These comparisons allow the derivation of smoothed calibration curves, which measure the (potentially non-linear) agreement between observed risks and model estimated risks,^7^ ^20^ across the entire range of predictions (ie, estimated risks from 0 to 1). The smoothed curve can be generated using, for example, polynomials, splines, or non-parametric methods,^43^ ^44^ ^45^ with confidence intervals derived post-estimation using methods such as Fisher’s Information or bootstrapping. The curve and confidence interval can be displayed on a calibration plot, as shown in figure 5 from an external validation of a model used to estimate five year recurrence risk after a primary breast cancer diagnosis.^5^ ^7^ The confidence interval (vertical range on the y axis) around the curve at a particular point on the x axis, provides the uncertainty interval for the actual risk of a group of individuals with the same estimated risk from the model (x axis). For example, figure 5 shows that the group of individuals with an estimated risk of 0.8 (x axis) have a 95% uncertainty interval around the curve (y axis) of between 0.78 and 1.00. Thus, if a new individual is estimated a risk of 0.8 by the model, we could use this interval to say: “In a group of 100 individuals with the same estimated risk as you, the model suggests that between about 78 and 100 will have a recurrence by five years.” The sample size of the evaluation study can be targeted to reach a particular level of precision in the calibration curve,^46^ ^47^ to reduce the width of these risk conditional uncertainty intervals.

A drawback is that, as these intervals are conditional only on the estimated risk, they do not take into account any other characteristics such as an individual’s specific predictor values; hence, the intervals are less individualised than those derived using the development itself (which are conditional on specific predictor values). To partly address this, calibration plots and curves can also be derived separately for subgroups defined by particular (combinations of) predictor values or other characteristics,^48^ for example, defined by age, sex, and ethnic group; this may also inform model fairness checks. However, as each subgroup will have a smaller sample size than the overall dataset, the uncertainty intervals around their calibration curves may be wide, unless the overall dataset is very large.^49^

Sometimes the uncertainty interval around the calibration curve may not even contain the corresponding risk estimate from the original model. For example, figure 5 shows that for individuals with an estimated risk of 0.2, the 95% confidence interval for this group’s actual risk is about 0.25 to 0.45 in the validation data. This value may be due to chance but could also be due to the original model being miscalibrated in the evaluation population (eg, due to a different case-mix or different predictor effects), which might motivate strategies to update and recalibrate the original model. As updating a model is akin to developing a new one, uncertainty distributions and intervals can then be derived by the methods explained in the previous section (eg, a bootstrap process).

## Concluding remarks

In summary, clinical prediction models enable an individual’s outcome risk to be estimated, but most only provide a point estimate of risk and do not present corresponding uncertainty intervals or distributions. We suggest that this should change, especially as many models are developed using an inadequately sized dataset leading to large model instability and large uncertainty in individual predictions. Presenting uncertainty of risk estimates helps stakeholders to evaluate and critically appraise a prediction model, and directs further research for developing and updating models, alongside other performance aspects (eg, calibration, discrimination, and clinical utility) and information detailed in the TRIPOD+AI reporting guideline.^42^

Derivation and display of uncertainty could be embedded in the same tool (eg, health system, web tool, or mobile app) that is used to apply the model to individuals.^50^ If appropriate, this uncertainty could be presented alongside point risk estimates within the doctor-patient consultation. However, communicating uncertainty of outcome risks with patients is challenging and should not always be done. Future research is needed into communicating prediction uncertainty, ideally with input from PPIE groups and clinical stakeholders because the best approach to disseminate and communicate uncertainty will often need tailoring to the setting and individual at hand.

**Summary points**

Clinical prediction models estimate an individual’s risk (probability) of a health related outcome to help inform patient counselling, and to support both patients and health professionals in making clinical decisions
Most models only allow a single point estimate of risk to be calculated; however, also providing the associated uncertainty (eg, via uncertainty distributions and intervals) gives a more complete picture
Quantifying the uncertainty of an individual’s risk provides an important model performance metric, which helps inform how that model should be used; shows the strength of evidence behind a model’s predictions; informs those critically appraising a model; contributes toward assessments of model fairness; and may enhance the doctor-patient conversation
In the model development dataset, uncertainty distributions and intervals can be derived for an individual’s risk using, for example, Bayesian or bootstrap approaches
At model evaluation, the confidence intervals of calibration curves can be used to express uncertainty of risk for a group of people with a particular estimated risk from the model
Effectively communicating uncertainty of outcome risks with patients is challenging and should not always be done; the best approach will often need tailoring to the clinical setting and individual at hand

## Web extra

Extra material supplied by authors

Web appendix: Extra material supplied by authors

## Notes

Funding: This paper presents independent research supported by an Engineering and Physical Sciences Research Council (EPSRC) grant for “Artificial intelligence innovation to accelerate health research” (number EP/Y018516/1), and by the National Institute for Health and Care Research (NIHR)’s Birmingham Biomedical Research Centre at the University Hospitals Birmingham NHS Foundation Trust and the University of Birmingham. GSC is supported by Cancer Research UK (CRUK; programme grant: C49297/A27294). PD is supported by CRUK (project grant: PRCPJT-Nov21\100021). LK is supported by an NIHR doctoral fellowship (NIHR303331) and by a core funding grant awarded to the CRUK clinical trials unit by CRUK (CTUQQR-Dec22/100006). MS is supported by the UK Research and Innovation AI programme, and the EPSRC, for CHAI-EPSRC AI Hub for causality in healthcare AI with real data (grant number EP/Y028856/1). Funding for FEHJ was provided by The Vanderbilt Institute for Clinical and Translational Research from a grant by the US National Center for Advancing Translational Sciences clinical translational science award programme (award number 5UL1TR002243-03). RDR, GSC, and AKD are NIHR Senior investigators. The views expressed are those of the author(s) and not necessarily those of the NHS, the NIHR, or the Department of Health and Social Care.

Patient and public involvement: In discussions with patient and public involvement and engagement groups in this and other projects (STANDING Together and Ewing sarcoma trials), participants provided opinions on the role of uncertainty of risk in clinical decision making, and whether this would be helpful to them in the patient-doctor consultation.

Contributors: RDR conceived the paper, and drafted an initial set of discussion points and examples with GSC and LA. All authors provided verbal or written comments, either at our main working group meetings, or within patient and public involvement and engagement group meetings on related projects, to help shape or balance the narrative and provide examples to illustrate the points in terms of statistical, clinical, patient and decision analysis reasoning. RDR, LA, and GSC then produced a final version of the paper, with further feedback over multiple iterations by all authors. After peer review, RDR revised the paper with feedback from LA and GSC, then from all authors. RDR is the guarantor. The corresponding author attests that all listed authors meet authorship criteria and that no others meeting the criteria have been omitted.

Provenance and peer review: Not commissioned; externally peer reviewed.
