# Supplementary material for: Uncertainty of risk estimates from clinical prediction models: rationale, challenges, and approaches
Source: BMJ. 2025 Feb 13;388:e080749. doi: 10.1136/bmj-2024-080749 (PMC12128882; doi:10.1136/bmj-2024-080749)
Supplement: Supplementary file 1 — Web appendix: Extra material supplied by authors [file rilr080749.ww.pdf]

Supplementary material

Supplementary material S1: decision analysis example

Fundamentally, the impact of uncertainty for an individual's risk is always context and individual specific, as it depends on the clinical problem, the outcome being predicted and what constitutes high risk for the individual at hand based on their utilities of particular outcomes and consequences. Consider a well-calibrated diagnostic prediction model has been published, in the form of a (logistic) regression equation that calculates a male patient's risk of having prostate cancer. If the equation calculates an individual's estimated risk to be 0.7, and the corresponding 95% uncertainty interval is 0.3 to 0.9, then the interval's range is wide but all consistent that the individual has a high risk and requires a biopsy. Conversely, if an individual's risk estimate was 0.005, but with a 95% uncertainty interval of 0.001 to 0.15, the upper range may still suggest biopsy is needed, even though the original point estimate was low. The impact of such uncertainty might be more relevant in a younger man (e.g., aged 40 years) with no comorbidities, for whom interventions may dramatically prolong life if prostate cancer is detected early, compared to an older man (e.g., 85 years) with many existing comorbidities.

It is helpful to formalise this process within a decision analysis framework. The decision is whether to biopsy (two possible actions: yes or no) and there are two possible states (prostate cancer present: yes or no), which creates four possible scenarios (pathways) shown in Figure S1. Assigned to each scenario is a "utility" (U1 to U4), which is a numerical measure that defines a value placed on a given pathway. These are specific to an individual, and essentially measure their preference for each scenario if it were known that the assumed state in that scenario was correct. The values of U1 to U4 are best considered relative to one another. For example, consider an individual expresses their utility of each pathway as: $U1_i = 100,\ U2_i = 5, U3_i = 0$ and $U4_i = 10$; this means they are expressing the action of biopsy if they do have prostate cancer ($U1_i = 100$), to be 10 times more important than the action of no biopsy if they do not have prostate cancer ($U4_i = 10$).

Figure S1 Summarising the four possible pathways that stem from the decision of whether or not to request a biopsy in an individual that may or may not have prostate cancer

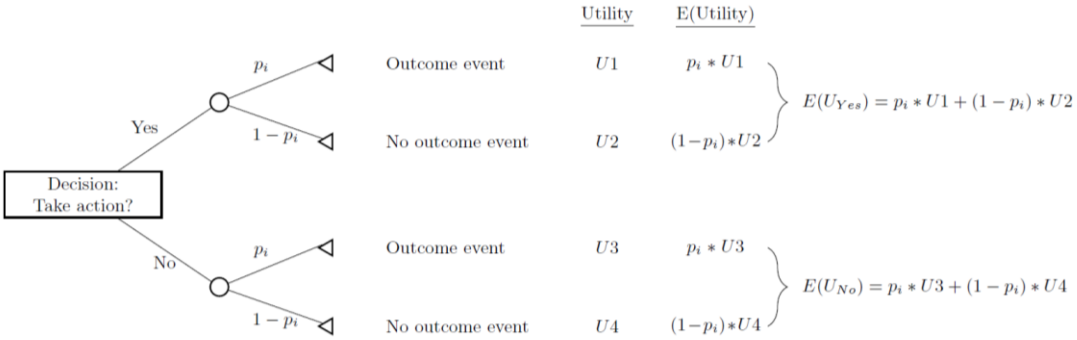

The individual's chosen utilities also define their risk threshold at which they would be willing to choose a biopsy. It corresponds to where the expected utility from biopsy ($E(U_{Biopsy})$) exceeds the expected utility from no biopsy (e.g., $E(U_{NoBiopsy})$):

$$E(U_{Biopsy}) > E(U_{No\ Biopsy})$$

This can be expressed in terms of the individual's probability (risk) of prostate cancer (p_i) weighted by the utility values:

$$(p_i * U1_i) + \left((1 - p_i) * U2_i\right) > (p_i * U3_i) + \left((1 - p_i) * U4_i\right)$$

Rearranging identifies the probability threshold at which the individual prefers a biopsy.[31]

$$p_{\text{THRESHOLD}i} > \left(1 + \frac{U1_i - U3_i}{U4_i - U2_i}\right)^{-1}$$

For example, let us return to the individual who expresses their utility of each outcome state as $U1_i = 100,\ U2_i = 5, U3_i = 0$ and $U4_i = 10$. Then:

$$p_{\text{THRESHOLD}i} > \left(1 + \frac{U1_i - U3_i}{U4_i - U2_i}\right)^{-1} = \left(1 + \frac{100 - 0}{10 - 5}\right)^{-1} = 0.048$$

If this individual's risk of prostate cancer was $\hat{p}_i = 0.051$, this suggests their preference is a biopsy, as their point estimate of risk exceeds their personal risk threshold, and so their expected utility of a biopsy is larger than their expected utility of no biopsy:

$$E(U_{Biopsy}) = (\hat{p}_i * U1_i) + \left((1 - \hat{p}_i) * U2_i\right) = (0.051 * 100) + \left((1 - 0.051) * 5\right) = 9.85$$

$$E\left(U_{No\ Biopsy}\right) = (\hat{p}_i * U3_i) + \left((1 - \hat{p}_i) * U4_i\right) = (0.051 * 0) + ((1 - 0.051) * 10) = 9.49$$

However, for this individual, **Error! Reference source not found.**(a) shows the uncertainty distribution for their risk, as obtained from the bootstrap process outlined in supplementary material S2, and it is quite skewed. Based on the 2.5% and 97.5% percentiles, the 95%

uncertainty interval for their risk is 0.016 to 0.09, but a large proportion of their distribution is < 0.05. The mean (median) of their uncertainty distribution is 0.047 (0.044), which is slightly lower and changes the expected risk to now being below the threshold of 0.05. Thus, the best estimate of the individual's risk has changed from 0.051 to 0.047 after accounting for uncertainty. The former is incorrect as it was derived assuming the prediction equation (logistic regression) was estimated with no uncertainty. Admittedly the difference is small, but the change is important here as it moves the best estimate from above to below the individual's risk threshold.

Error! Reference source not found.(b) shows the corresponding uncertainty distribution for $U_{Biopsy} - U_{No\ Biopsy}$, and the mean (median) is -0.11 (-0.43), which is below zero and so in favour of no biopsy. Again, this is the opposite to when just considering their point estimate of risk derived ignoring uncertainty. Hence, in this example, deriving the individual's expected risk and utility directly from their uncertainty distribution (rather than the fixed logistic regression equation) has changed the individual's decision from biopsy to no biopsy.

Supplementary material S2: The bootstrap process for deriving uncertainty distributions

Figure S2: The bootstrap process to examine instability and derive uncertainty intervals and distributions of model predictions in a chosen target population, as adapted from Riley and Collins.[12]

APPROACH: After developing a model using a dataset of n participants from the chosen target population, the bootstrap process to examine instability follows a seven-step process, as follows:

- Step 1: Use the developed model to estimate risk ($\hat{p}_i$) for each individual ($i = 1$ to n) in the development dataset.
- Step 2: From the development dataset, generate a bootstrap sample with replacement, of size n.
- Step 3: Develop a prediction model in the bootstrap sample, replicating exactly (or as far as practically possible) the same model development approach and set of candidate predictors as used originally.
- Step 4: Use the bootstrap model developed in step 3 to make predictions for each individual (i) in the original dataset. We refer to these predictions as $\hat{p}_{bi}$, where b indicates which bootstrap sample the model was generated in (b = 1 to B, where B gives the number of bootstrap iterations).
- Step 5: Repeat steps 2 to 4 a total of $(B - 1)$ times (with B at least 200 in total).
- Step 6: Store all the predictions from the B iterations of steps 2 to 5 together in a single dataset, containing for each individual a prediction ($\hat{p}_i$) from the original model and B predictions ($\hat{p}_{1i}$, $\hat{p}_{2i}$, ... , $\hat{p}_{Bi}$) from the bootstrap models.
- Step 7: Summarise the uncertainty in predictions, by plotting a prediction instability plot (a scatter of the B predicted values for each individual against their original predicted value) and by calculating uncertainty distributions (the distribution of the original and B predicted values) and uncertainty intervals (e.g., a 95% interval based on the 2.5% and 97.5% values) for each individual. If risk thresholds are used to decide a particular clinical action (e.g., initiate treatment if an individual's point risk estimate ≥ 0.05), a classification instability plot could be calculated, displaying the proportion of each individual's uncertainty distribution that falls in the opposite side of the risk threshold than their point estimate.

NOTES:

- To calculate uncertainty of risks for individuals outside the development dataset, the B bootstrap models from the above process need to be stored (alongside the original model) so that they can be applied to obtain B predicted values for any new individual, which are then used to derive uncertainty distributions and intervals as in Step 7.
- Crucially, the model development process must target well-calibrated predictions (i.e., estimated and observed risks should agree, ideally across the full spectrum of risks from 0 to 1), otherwise the uncertainty intervals will reflect uncertainty of predictions that are poorly calibrated. Calibration will often require further assessment in new data from the target population, especially if the model development dataset was small.
